# Stage-specific efficacy of pharmacological interventions on gait impairments in Parkinson’s disease

**DOI:** 10.3389/fneur.2025.1657884

**Published:** 2025-10-15

**Authors:** Jun Zhang, Le Chen, Jun Li

**Affiliations:** ^1^Department of Neurology, Beijing Tsinghua Changgung Hospital, School of Clinical Medicine, Tsinghua Medicine, Tsinghua University, Beijing, China; ^2^Clinical Research Center, Xuanwu Hospital Capital Medical University, Beijing, China

**Keywords:** Parkinson’s disease, gait disorders, gait domain, video capture technology, advanced PD

## Abstract

**Objective:**

This study aims to clarify the variations and the therapeutic effects of medicine on gait by analyzing the gait characteristics before and after medication in various stages of Parkinson’s disease (PD).

**Methods:**

This prospective study included 60 patients with PD [Hoehn-Yahr (H-Y) stage 1–4] at the department of Neurology of Beijing Tsinghua Changgung Hospital, and 30 gender and age - matched healthy controls. The ReadyGo system was used to record gait parameters. The levodopa challenge test was applied to assess the therapeutic effect of medicine on gait.

**Results:**

We observed a shorter stride length and stride height, a longer stride time and turn-around time, and a reduction of stride speed in the PD group compared with the healthy control group significantly. No significant changes were noted in the variability of stride parameters (stride frequency variability and stride length variability). The radar chart demonstrated a gradual decline in gait parameters as the disease progressed. Compared to healthy control group, significant differences in stride speed and stride time were observed from H-Y stage 1 (*p* < 0.05) while stride length, stride width, and turn-around time were from H-Y stage 2 (*p* < 0.05), and step height is H-Y stage 3 (*p* < 0.05). After administering levodopa, there was a marked improvement in stride speed (*p* = 0.009) and turn-around time (*p* = 0.005). The most notable improvements in stride speed and turn-around time occurred at H-Y stage 3. Improvements in non-gait domains were more notable across all stages of PD patients than gait domains.

**Conclusion:**

Gait changes can serve as a new early diagnosis marker for PD because of the early significant change in gait parameters especially on stride speed and stride time. As the disease advances, various gait parameters gradually deteriorate, suggesting that objective gait monitoring can provide a reference method to dynamically observe PD progression. Unlike the previous view that medicine has limited effect on gait, this study found that although the effect of medicine on gait is not as remarkable as that on tremor and rigidity, medicine can still effectively improve gait, especially in the patients with advanced PD.

## 1 Introduction

Parkinson’s disease (PD) is a common neurodegenerative disorder characterized by chronic and progressive impairment of the central nervous system. The incidence of this disease increases with advancing age and it reaches an estimated 200 cases per 100,000 individuals aged 65 years and older (1), imposing substantial economic burdens on affected patients and their families. A key clinical feature of PD is gait disorder, which can manifest early in the disease course and progressively worsen over time. Gait disorder significantly affects patients’ quality of life and increases the risk of falls, fractures, and potentially life-threatening events (2, 3). Patients with PD typically exhibit gait alterations marked by reduced walking speed, shortened stride length, and prolonged step duration (4). Past researches suggest that pharmacological treatments have limited effectiveness in gait related symptoms (5). However, the past clinical assessments of gait in PD patients were mostly based on the clinicians subjective judgment or the relevant scales evaluation. These methods were not sensitive to the changes of gait disorders in the progression of the disease and were highly subjective. Therefore, the past viewpoints about gait changes in PD and the limited therapeutic effect of medicine on gait disorder mentioned above were mainly based on subjective evaluations or relevant scales, which need to be further evaluated by objective methods. With the development of motion analysis technology, the use of video capture technology to quantify gait parameters has been more and more widely carried out in clinical work and research (6–8), providing an objective means to verify those past viewpoints on gait changes in PD.

Based on this, gait parameters were divided into three gait domains: spatial parameters, temporal parameters and parameters of variation. In this research, our target is to clarify the characteristics of early gait changes in PD patients and the changes of gait impairment in different stages of disease progression by detecting the gait parameters of PD patients in different Hoehn-Yahr (H-Y) stages, and determine the effect of medicine on gait during the progression of PD.

## 2 Materials and methods

### 2.1 Participants

We recruited 60 patients who were admitted to the Neurology Department of Beijing Tsinghua Changgung Hospital from January 2024 to September 2024. The inclusion criteria were the following: (1) diagnosed as an idiopathic PD according to the International Parkinson’s Disease and Movement Disorder Society (MDS) clinical diagnostic criteria (9); (2) H-Y grade ≤4; (3) Mini Mental State Examination (MMSE) score >24. The exclusion criteria were the following: (1) patients with secondary Parkinson’s disease or Parkinson’s plus syndrome; (2) suffering from other nervous system diseases or serious medical diseases such as heart, liver, lung, kidney, etc.; (3) freezing of gait identified by the evaluator or the device during walking during gait assessment by the wearable device; (4) those who failed to cooperate with clinical evaluators; (5) patients with orthopedic or musculoskeletal disease or other causes that may affect balance or gait (e.g., eye disease, etc.).

Healthy controls were partners of PD patients or volunteers. The inclusion criteria were as follows: (1) no neurological, ophthalmic, orthopedic, or musculoskeletal disease that could affect balance or gait (e.g., eye diseases, etc.); (2) no heart, liver, lung, kidney and other medical diseases; (3) Mini-Mental State Examination (MMSE) score >24.

This was a prospective study. Randomized control matching was performed. It was approved by the Ethics Committee of Beijing Tsinghua Changgung Hospital (Approval number: 23402-4-02). Written informed consent was obtained from all the participants according to the Declaration of Helsinki. All methods were performed in accordance with the relevant guidelines and regulations.

### 2.2 Methods

(1) General information collection: The clinical information of PD patients was collected, including age, gender, disease duration, Unified Parkinson’s Disease Rating Scale (UPDRS) score, H-Y stage, MMSE score. We calculated the total daily levodopa equivalent dose according to the anti-PD drug use condition (10). The age, gender and MMSE score of the healthy control group were collected.
(2) Assessment of gait data: Gait parameters were assessed using a quantitative motor function assessment system (ReadyGo, Beijing Keruiyi Information Technology Co., Ltd.). The ReadyGo system uses only a set of cameras, including an RGB (red/green/blue) camera and a single-depth camera, to capture three-dimensional (3D) motion and perform depth learning for skeletal point localization. The device can uniquely track multiple bones by observing and estimating 3D joints and landmarks through depth vision perception and eliminates the need for subjects to wear any sensors. For kinematic analysis of gait parameters, participants were asked to walk 3 round trips on a 3-meter pavement at their usual speed without the use of any walking aid.
(3) Levodopa Challenge Test (LCT): Participants discontinued their intake of levodopa and other anti-Parkinson’s disease medications 12 h before the test. The trial drug was madopar (250 mg per tablet, contained levodopa 200 mg and benserazide 50 mg, Roche), and the dose was converted to 1.5 times the levodopa equivalent dose of the first anti-PD drug taken each morning. For participants who had not previously taken any anti-PD medications, a half-tablet of madopar was administered orally (11). Baseline assessments, including UPDRS-III and gait scores, were recorded while participants were fasting. Following this, participants ingested madopar. UPDRS-III and gait scores were then assessed hourly after levodopa administration until 3 h after taking the drug. The maximum improvement rate (MIR) was determined using the formula: [(pre-levodopa baseline score - lowest post-levodopa score)/pre-levodopa baseline score] × 100%. Our team has appointed a dedicated gait assessment specialist, who is blinded to the “ON” and “OFF” medication states of PD patients.
(4) In the PD staging system, the H-Y staging method was utilized. For comparative analysis, stages 1.5 and 1 were categorized into the stage 1 group; stages 2 and 2.5 were grouped into the stage 2 group; and stages 3 and 4 were classified into the stage ≥3 group.
(5) Gait parameters and non-gait parameters: In this study, gait parameters included spatial metrics (such as stride length, stride width, and stride height), temporal metrics (including stride speed, stride time, and turn-around time), and variability metrics (like stride length variability and stride frequency variability). The degree of variation was defined as the ratio of variance to the mean. The non-gait score was obtained by summing the scores for items 18 through 26 on the UPDRS rating scale. Both the maximum improvement rate of the non-gait score and the improvement rate of the gait parameter scores were determined using the previously mentioned formula for calculating the maximum improvement rate.

### 2.3 Statistics

SPSS Statistics 26.0 (IBM, United States) was utilized for data analysis. For continuous variables that followed a normal distribution, results were expressed as mean ± standard deviation ( $\bar{x}$ ±s). Continuous variables that did not follow a normal distribution were described using median (interquartile range) [Med (Q1, Q3)]. Categorical variables were presented as frequencies and percentages. To compare age and MMSE scores between the Parkinson’s disease (PD) group and the healthy control group, an independent samples t-test was applied. Gender differences between the two groups were evaluated using the χ^2^ test.

Seven gait variables were collected, including stride length, stride width, stride height, stride speed, stride time, turn-around time, stride length variation and stride frequency variation. To evaluate the consistency of variance for these gait characteristics between the healthy control group and the PD group, Levene’s test was conducted. The normality of the data was assessed using histograms and Q-Q plots. For data that satisfied both normal distribution and homogeneity of variance, one-way analysis of variance (ANOVA) was utilized for inter-group comparisons. In cases where homogeneity of variance was not met but normal distribution was maintained, Welch’s ANOVA was applied. The Kruskal-Wallis H test was used for comparing gait variables that did not conform to a normal distribution. Post-hoc pairwise comparisons were conducted using the Bonferroni t-test for groups with homogeneous variances or the Games-Howell test for those with heterogeneous variances. Gait metrics were converted into Z-scores, referencing the healthy controls [for instance, stride Z-score = (individual stride - mean stride of healthy controls)/standard deviation of strides in healthy controls], to create radar charts. These charts facilitated the comparison of the extent of gait parameter impairment in PD patients across different stages of the disease.

The differences of gait parameters between PD patients and healthy controls were compared by independent sample t test, and the differences of age and MMSE in different stages of PD patients were compared by ANOVA test. The differences of gait parameters between different stages of PD patients were compared by one-way analysis of variance, and the differences of gait parameters before and after drug treatment in PD group were compared by group t test.

We used Spearman to analysis the correlations between gait parameters and H-Y stages, and Pearson’s for the correlation between UPDRS-III scores and the MIR of gait parameters.

## 3 Results

### 3.1 Demographic and clinical data of healthy controls and Parkinson’s disease patients

A total of 30 participants were recruited for the healthy control group, consisting of 14 males and 16 females, with a mean age of 64.90 ± 5.81 years. The PD group included 60 patients, comprising 29 males and 31 females, with an average age of 67.22 ± 8.87 years. The PD group had a mean disease duration of 3.28 ± 2.75 years, a mean UPDRS-III score of 23.73 ± 14.31, and an average H-Y stage of 2.18 ± 0.83. Specifically, within the PD cohort, 20% (*n* = 12) were at H-Y stage 1, 6.67% (*n* = 4) at H-Y stage 1.5, 35% (*n* = 21) at H-Y stage 2, 5% (*n* = 3) at H-Y stage 2.5, 28.33% (*n* = 17) at H-Y stage 3, and 5% (*n* = 3) at H-Y stage 4. There were no statistically significant differences between the two groups in terms of gender distribution, age, or MMSE scores (all *p* > 0.05; Table 1).

**Table 1:** The clinical data of control group and Parkinson′s disease group.

Clinical data	PD group (*N* = 60)	Control group (*N* = 30)	*p*-value
Age (years)	67.22 ± 8.87	64.90 ± 5.81	0.142
Gender (male/female)	29/31	14/16	0.881
Disease duration (years)	3.28 ± 2.75	/	/
Hoehn and Yahr grade	2.18 ± 0.83	/	/
Mini Mental Status Examination	27.40 ± 1.50	27.63 ± 1.35	0.470
UPDRS-III	23.73 ± 14.31	/	/

### 3.2 Differences in gait parameters between the Parkinson’s disease group and the healthy control group

The differences in various gait parameters between healthy controls and PD patients are presented in Table 2. The analysis revealed that PD patients exhibited reductions in stride length, stride speed, and stride height, while showing increases in stride time, stride width, and turn-around time. These differences were statistically significant (*p* < 0.05). However, no significant differences were observed in the variability of stride frequency and stride length.

**Table 2:** Comparison of parameters between PD group and control group.

Parameter	PD group (*N* = 60)	Control group (*N* = 30)	*p*-value
Stride length	0.89 ± 0.24	1.19 ± 0.27	0.000
Stride width	0.14 ± 0.02	0.12 ± 0.01	0.000
Stride height	0.10 ± 0.03	0.11 ± 0.02	0.010
Stride speed	0.79 ± 0.23	1.56 ± 0.22	0.000
Stride time	1.12 ± 0.18	0.72 ± 0.08	0.000
Turn-around time	2.37 ± 2.42	0.75 ± 0.20	0.000
Stride frequency variation	0.12 ± 0.04	0.19 ± 0.26	0.161
Stride length variation	16.86 ± 11.18	13.20 ± 10.85	0.184

### 3.3 Characteristics of gait impairment across different Hoehn-Yahr stages in PD

**Figure 1:**
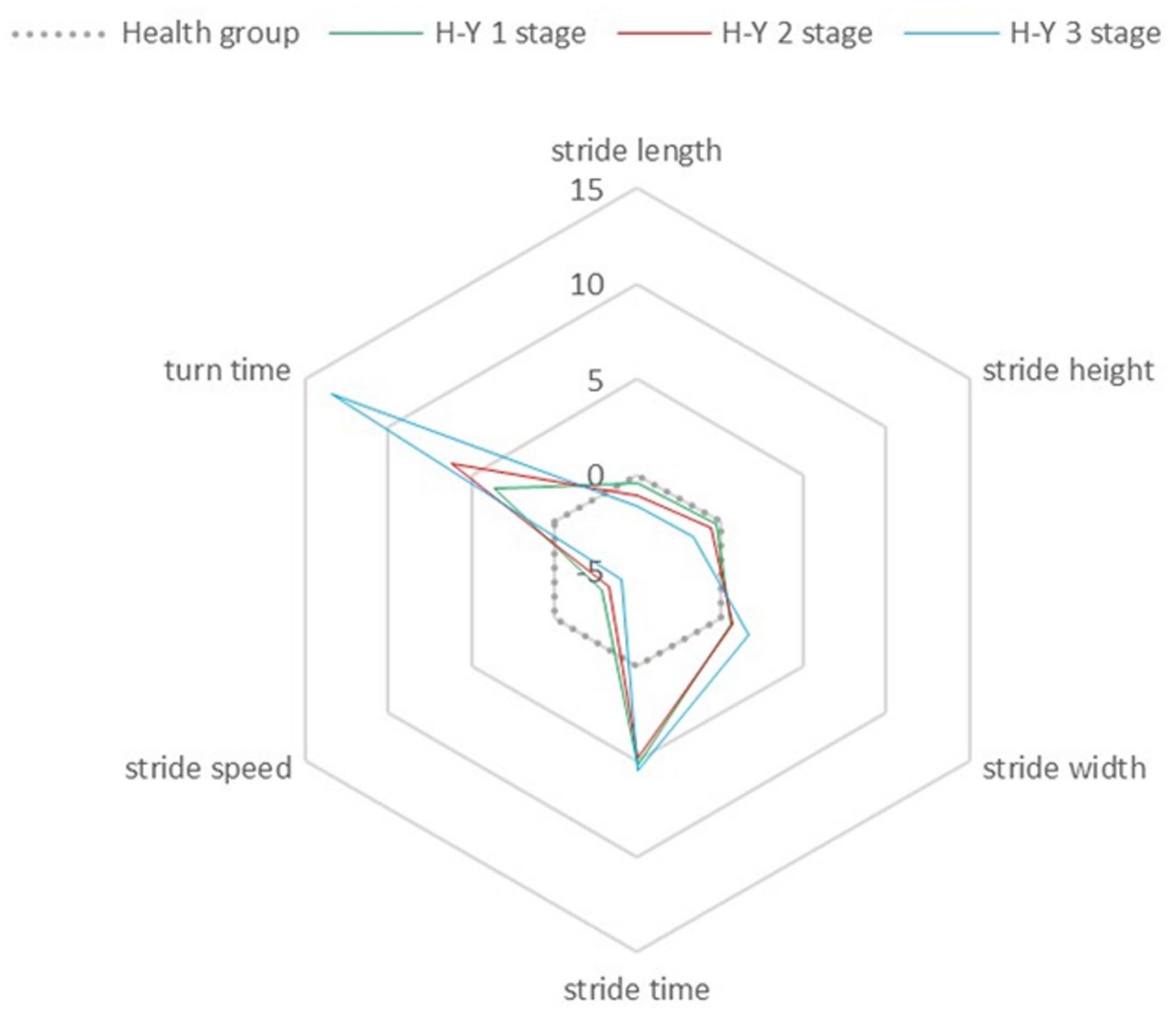
The distinctions in gait parameters between PD patients at different H-Y stages and healthy group.

Table 3 provides a summary of the differences in age and cognitive function across PD groups at varying H-Y stages. Table 4 and Figure 1 emphasize the distinctions in gait parameters between PD patients at different H-Y stages and healthy individuals, as well as among PD patients across these stages. Table 5 depicts the correlations between gait parameters and H-Y stages. The findings can be summarized as follows: (1) the age and cognitive abilities of PD patients across various H-Y stages were well-matched. (2) As the disease advances, gait parameters in each domain progressively worsen. The stride length, stride speed and stride height of PD patients were negatively correlated with the H-Y, and stride width and turn-around time were positively correlated. Compared to the healthy control group, changes in stride speed and stride time became apparent at H-Y stage 1. Significant differences in stride length, stride width, and turn-around time emerged from H-Y stage 2 onwards. Notable variations in step height were observed starting at H-Y ≥ 3. Therefore, temporal parameters were found to be the most sensitive indicators, showing abnormalities even in the early stages of the disease.

**Table 3:** Comparison of age and MMSE among different H-Y stages.

Stage	N	Age	MMSE
H-Y 1	12	61.58 ± 8.70	27.67 ± 1.50
H-Y 1.5	4	69.75 ± 9.14	28.25 ± 1.26
H-Y 2	21	68.71 ± 7.52	27.52 ± 1.47
H-Y 2.5	3	70.33 ± 10.12	27.33 ± 2.31
H-Y 3	17	67.29 ± 9.01	26.76 ± 1.35
H-Y 4	3	72.33 ± 13.43	28.00 ± 2.00
F		1.486	1.078
P		0.210	0.383

H‑Y, Hoehn‑Yahr.

**Table 4:** Comparison of gait parameters between healthy group and PD group.

N	Healthy 30	H-Y 1 16	H-Y 2 24	H-Y ≥ 3 20	F	*p*
Stride length	1.19 ± 0.27	1.06 ± 0.12	0.90 ± 0.22^*^	0.74 ± 0.23^*^	18.025	<0.001
Stride width	0.12 ± 0.01	0.13 ± 0.02	0.14 ± 0.02^*^	0.15 ± 0.02^*^	9.948	<0.001
Stride height	0.11 ± 0.02	0.11 ± 0.01	0.10 ± 0.03	0.09 ± 0.02^*^	6.734	<0.001
Stride speed	1.56 ± 0.22	0.92 ± 0.15^*^	0.82 ± 0.21^*^	0.65 ± 0.23^*^	94.651	<0.001
Stride time	0.72 ± 0.08	1.12 ± 0.10^*^	1.09 ± 0.16^*^	1.14 ± 0.24^*^	45.637	<0.001
Turn-around time	0.75 ± 0.20	1.48 ± 0.58	2.01 ± 1.70^*^	3.50 ± 3.49^*^	8.804	<0.001

H‑Y, Hoehn‑Yahr; ^*^Significant differences compared with healthy group (*p* < 0.05).

**Table 5:** The correlations between gait parameters and H-Y stages.

Parameter	Correlation coefficients	*p*
Stride length	−0.570	<0.001^*^
Stride width	0.330	0.010^*^
Stride height	−0.392	0.002^*^
Stride speed	−0.572	<0.001^*^
Stride time	0.047	0.722
Turn-around time	0.438	<0.001^*^

^*^The mean difference is significant at the 0.05 level.

### 3.4 The impact of pharmacological interventions on gait parameters in PD patients across various Hoehn-Yahr stages

The outcomes of the improvement in each gait parameter of PD group after administering levodopa are summarized in Table 6, and the correlation between UPDRS-III scores of PD patients and the MIR of gait parameters are presented in Table 7. Given the significant differences in the rates of improvement in stride speed and turn-around time before and after treatment, a further comparison of these improvement rates was conducted across different H-Y stages (Table 8). These results suggest that there was a notable enhancement in stride speed and turn-around time following levodopa treatment. The most substantial improvement rates for stride speed and turn-around were observed in patients at H-Y ≥ 3 stage, which matches with Table 7, that is the MIR of gait domains is positively correlated with UPDRS-III scores.

**Table 6:** The gait parameter improvement in PD group before and after levodopa.

Parameter	PD group (*N* = 60)		*p*
	Pre	Post	
Stride length	0.89 ± 0.24	0.93 ± 0.25	0.089
Stride width	0.14 ± 0.02	0.14 ± 0.02	0.337
Stride height	0.10 ± 0.03	0.10 ± 0.02	0.072
Stride speed	0.79 ± 0.23	0.83 ± 0.22	0.009^*^
Stride time	1.12 ± 0.18	1.12 ± 0.17	0.852
Turn-around time	2.37 ± 2.42	2.01 ± 1.73	0.005^*^

^*^Significant differences compared with healthy group (*p* < 0.05).

**Table 7:** The correlation of MIR of gait domains and UPDRS-III scores.

Parameter	Correlation coefficients	*p*
Stride length	0.346	<0.001^*^
Stride width	−0.023	0.861
Stride height	0.293	0.023^*^
Stride speed	0.522	<0.001^*^
Stride time	0.227	0.081
Turn-around time	0.238	0.067

^*^The mean difference is significant at the 0.05 level.

**Table 8:** MIR of non-gait domain and gait domain in PD.

Parameter	H-Y 1 *N* = 16	H-Y 2 *N* = 24	H-Y 3 *N* = 20	F	*p*
Non-gait domains	0.49 ± 0.23	0.36 ± 0.22	0.41 ± 0.18	1.851	0.166
Stride length	0.08 ± 0.17^c^	0.10 ± 0.10^c^	0.24 ± 0.33^ab^	3.179	0.049
Stride width	0.03 ± 0.04	0.05 ± 0.04	0.04 ± 0.04	0.652	0.525
Stride height	0.10 ± 0.07^c^	0.08 ± 0.08^c^	0.20 ± 0.23^ab^	4.229	0.019
Stride speed	0.07 ± 0.13^c^	0.11 ± 0.11^c^	0.28 ± 0.27^ab^	6.887	0.002
Stride time	0.04 ± 0.05	0.04 ± 0.04	0.07 ± 0.11	1.606	0.210
Turn-around time	0.13 ± 0.16^c^	0.14 ± 0.14^c^	0.25 ± 0.19^a^^,^^b^	3.243	0.046

a Significant differences compared with H-Y 1 stage (*p* < 0.05).

b Significant differences compared with H-Y 2 stage (*p* < 0.05).

c Significant differences compared with H-Y 3 stage (*p* < 0.05).

### 3.5 Comparison of medicine efficacy in non-gait and gait domains among patients with PD

The disparity in improvement between gait and non-gait domains among PD patients is detailed in Tables 8, 9. Table 8 illustrates the trends in non-gait improvement rates and gait parameter improvement rates across different H-Y stages of PD. The findings suggest that (1) Across all PD patients, improvements in non-gait domains are more pronounced compared to those in gait domains, indicating that pharmacological interventions yield better outcomes in non-gait aspects than in gait. (2) As the disease progresses, the efficacy of medication on non-gait symptoms has no change, whereas its effectiveness on gait parameters increases.

**Table 9:** MIR of non-gait domain and gait domain in PD.

Parameter	PD group (*N* = 60)	
	MIR	*p*
UPDRS-III	0.41 ± 0.21	
non-gait domains	0.41 ± 0.22	2.301
stride length	0.14 ± 0.23	0.000*
stride width	0.04 ± 0.04	0.000*
stride height	0.13 ± 0.15	0.000*
stride speed	0.16 ± 0.21	0.000*
stride time	0.05 ± 0.08	0.000*
turn-around time	0.17 ± 0.17	0.000*

*Significant differences compared with maximum improvement rate of UPDRS-III. MIR: maximum improvement rate.

## 4 Discussion

This study used an objective, video-based motion capture system to quantitatively analyze gait parameters in PD patients at different H-Y stages, both before and after levodopa administration. To our understanding only a few reports have objectively explored the efficacy of levodopa on gait abnormalities across various stages of PD by gait analysis technology. Our key innovations are threefold: First, we found that specific temporal parameters were the most sensitive parameters for PD gait impairment, and these parameters were abnormal in the early stage of the disease. Second, we showed that quantitative gait analysis can reflect disease progression over time, with spatial parameters worsening significantly from H-Y stage 2 onward. Third, we challenge the common belief that levodopa has limited effects on gait, by objectively demonstrating its significant improvements in stride speed and turn time, especially in advanced-stage (H-Y ≥ 3) patients. These insights were enabled by our stage-specific design and the use of high-resolution, sensor-free motion capture, which provides a more detailed and objective gait assessment than traditional rating scales.

With disease progression, each gait parameter shows a gradual decline. During the early phase, alterations in stride speed and stride time are especially prominent. This may because stride speed and stride time are more dependent on the regulation of gait automation and rhythm by the basal ganglio-cortical circuit, which is impaired in the early stage of PD (3). Temporal parameters may more directly reflect core deficits in motor initiation and rhythm maintenance. Significant differences in spatial gait parameters only became apparent after H-Y 2 stage, indicating that these parameters may not be suitable for distinguishing early-stage PD from healthy controls but are more appropriate for evaluating PD progression. These results are consistent with prior studies, which suggest that early-stage PD patients mainly experience diminished swing amplitude, progressing to freezing and panic gait in later stages (12). No significant differences were observed between healthy controls and PD patients regarding stride variability and stride frequency variability. Although variability has traditionally been considered a predictor of falls, this study did not demonstrate a difference between PD patients and healthy controls. It is probably due to that this study mainly includes H-Y ≤ 3 stage patients, with few stage 4 patients and no stage 5 patients who are at the highest risk of falling. Consequently, this suggests that variability parameters may not be reliable indicators for the early detection of gait abnormalities. This study indicates changes in gait parameters, particularly temporal parameters, can serve as early indicators for disease diagnosis. Early-stage PD patients exhibit gait abnormalities characterized by decreased stride speed and extended stride duration. They did not show typical panic gait features such as reduced stride length or increased turning time. Reduced walking speed and increased stride duration are common in normal older adults, proposing the difficulty of identifying early gait changes in PD for either doctors or family observations, which highlights the need for objective gait measurement technologies rather than human evaluation to detect abnormal patterns promptly and enables timely intervention.

Currently, the underlying mechanisms of gait disorders in PD are not yet fully understood. Under normal conditions, the manifestation of gait primarily involves three processes: the gait automation process, the integration process of peripheral sensory information, and the cognitive process (13). Firstly, the gait automation process largely depends on subcortical structures, including the basal ganglia and the brainstem. In patients with PD, damage to the dopaminergic (DA) neurons in the substantia nigra pars compacta projects to the basal ganglia nuclei. This leads to increased inhibition of the thalamus and pontine nuclei, and a reduction in the excitability of the motor structures within the cerebral cortex (14), thereby influencing the gait automation process. In the early stage of PD, gait disorders are often attributed to the loss of DA neurons in the substantia nigra pars compacta. This loss results in abnormal motor function of the basal ganglia, disrupting the gait automation process. Consequently, PD patients exhibit gait alterations such as a reduced amplitude of arm swing, shorter strides, and a slower walking speed. This finding is consistent with what we observed in the H-Y 1 and 2 groups of our study. Secondly, PD patients may experience varying degrees of damage to the thalamocortical system, basal ganglia nuclei, and cerebral cortex. This can lead to decreased attention, impaired sensory-motor integration, and a reduced ability to compensate for gait control through active movement, thus increasing gait variability (15). As the disease progresses, the information integration and cognitive processes in PD patients gradually deteriorate, and the function of the basal ganglia further declines, exacerbating gait disorders. Furthermore, some research indicates that emotional state is also one of the factors contributing to gait disorders (16, 17). Research has revealed that gait disorders in PD are not solely associated with the impairment of dopaminergic neurons but also involve functional abnormalities in other neural pathways, such as the basal ganglia-thalamus-cortical loop and the cerebellum-brainstem-spinal cord pathway (3). Dopamine can enhance dopaminergic neural function; however, its regulatory influence on other non-dopaminergic pathways is restricted. Consequently, its efficacy in improving gait is relatively modest. Levodopa is transformed into dopamine within the brain and primarily acts on dopamine receptors, directly alleviating muscle rigidity and bradykinesia. Nevertheless, gait control necessitates the coordinated interplay of multiple brain regions and neurotransmitter systems. Levodopa is unable to comprehensively modulate these intricate neural mechanisms, resulting in a relatively less pronounced improvement in gait compared to non-gait symptoms. This mechanism accounts for the finding in this study that the ameliorative effect of levodopa on the gait domain is less remarkable than that on the non-gait domain.

Moreover, modifications in neurotransmitter systems, such as cholinergic and noradrenergic non-dopaminergic neural networks, also play a crucial role in the development of gait abnormalities (18). In the early stage of PD, gait abnormalities are generally mild and mainly linked to the degeneration of dopaminergic neurons (19). This neuronal degeneration predominantly affects the sensorimotor striatum located in the posterior putamen, which results in compromised motor automation. Nevertheless, attentional control mechanisms in the initial phase can partly offset these functional impairments (19). In the mid-to-late stage of PD, the severe degeneration of dopaminergic neurons and the decline of non-dopaminergic neurons are involved (20). This potentially leads both clinicians and patients to view the medication as ineffective methods for gait problems in advanced PD incorrectly. Our analysis indicates that levodopa is more effective in enhancing gait for PD patients in advanced stages compared to those in the early. This finding implies that while dopaminergic neurons are substantially damaged in advanced PD, dopamine supplementation can successfully increase the levels in the brain, thereby alleviating gait problems. This could potentially be attributed to the fact that patients in the advanced stage of PD typically administer higher dosages of dopaminergic medications. As a result, they are more likely to reach the threshold necessary for improving certain gait parameters. Furthermore, in advanced stage patients, the cortical compensatory capacity may deteriorate. Consequently, their gait performance becomes more directly reliant on the residual functionality of the dopaminergic system. Even though the functional state of the dopaminergic system remains relatively low during this period, pharmacological interventions may still yield a relatively more notable “absolute” improvement. Therefore, our research suggests that for advanced PD patients, drug adjustment should not be abandoned prematurely, as it may be effective in improving their symptoms. Nevertheless, it is crucial to recognize that various non-dopaminergic factors also play a role in gait disorders. Even with improvements from levodopa therapy, significant gait issues may remain.

## 5 Conclusion

This study highlights the potential of quantitative gait temporal parameters, specifically gait speed and stride time, as biomarkers for the early detection and monitoring of disease progression. Levodopa has been shown to significantly improve stride speed and reduce turn-around time, particularly in patients with advanced PD. Critically, the quantitative gait analysis methodology established herein—utilizing accessible video capture technology—provides a robust, scalable, and objective framework for future research. This framework is not only applicable for investigating broader PD gait abnormalities but also holds significant promise for elucidating the pathophysiology and treatment responses of specific conditions like freezing of gait (FOG) in appropriately designed studies. We recommend that subsequent research leverage such objective tools to overcome the limitations of subjective scales and further refine our understanding of gait impairment in PD.

However, several limitations should be acknowledged. The sample size was relatively small, which may have reduced the statistical power for certain gait parameters. Furthermore, the recruitment of patients at H-Y 4–5 stages was limited due to the increased risk of falls associated with these stages. Consequently, the ability to evaluate drug-specific effects on FOG, which predominantly occurs in mid (H-Y 3–4) to late (H-Y5) stage, was constrained. Only two patients in this study were confirmed to exhibit FOG; therefore, the impact of pharmacological intervention on FOG could not be analyzed. Numerous factors influence gait performance in PD patients, including emotional states and types of anti-Parkinsonian medications. Although patients were categorized based on H-Y stage, this classification system is relatively coarse and may result in significant within-group heterogeneity.

To enhance the accuracy and comprehensiveness of future research, these limitations must be addressed. Expanding recruitment to multiple clinical centers would ensure a more diverse and representative patient population. Increasing the sample size would also improve statistical reliability and generalizability. Future studies should specifically enroll a sufficient number of patients with FOG and employ standardized assessment tools to directly evaluate the efficacy of pharmacological interventions on this critical symptom. Refining the analysis of gait variables and incorporating relevant confounding factors-particularly emotional states-would contribute to the development of more robust gait models. Comparative analyses of different anti-Parkinsonian medications across disease stages could provide valuable insights into optimizing treatment strategies for PD patients. Additionally, incorporating the UPDRS as a core assessment tool would facilitate more sensitive and comprehensive analyses of clinical-gait correlations.

## Data Availability

The original contributions presented in the study are included in the article/supplementary material, further inquiries can be directed to the corresponding author.

## References

[1] Tanner CM Ropper AH Ostrem JL. Parkinson’s disease. N Engl J Med. (2024) 391:442–52. doi: 10.1056/NEJMra2401857 39083773

[2] Del Din S Elshehabi M Galna B Hobert MA Warmerdam E Suenkel U. Gait analysis with wearables predicts conversion to Parkinson disease. Ann Neurol. (2019) 86:357–67. doi: 10.1002/ana.25548, PMID: 31294853 PMC6899833

[3] Mirelman A Bonato P Camicioli R Ellis TD Giladi N Hamilton JL. Gait impairments in Parkinson's disease. Lancet Neurol. (2019) 18:697–708. doi: 10.1016/S1474-4422(19)30044-4, PMID: 30975519

[4] Horak FB Mancini M Carlson-Kuhta P. Balance and gait represent independent domains of mobility in Parkinson disease. Phys Ther. (2016) 96:1364–71. doi: 10.2522/ptj.20150580, PMID: 27034314 PMC5009185

[5] Smulders K Dale ML Carlson-Kuhta P Nutt JG Horak FB. Pharmacological treatment in Parkinson's disease: effects on gait. Parkinsonism Relat Disord. (2016) 31:3–13. doi: 10.1016/j.parkreldis.2016.07.006, PMID: 27461783 PMC5048566

[6] Franco A Russo M Amboni M Ponsiglione AM Di Filippo F Romano M. The role of deep learning and gait analysis in Parkinson’s disease: a systematic review. Sensors. (2024) 24:5957. doi: 10.3390/s24185957, PMID: 39338702 PMC11435660

[7] Lam WWT Tang YM Fong KNK. A systematic review of the applications of markerless motion capture (MMC) technology for clinical measurement in rehabilitation. J Neuroeng Rehabil. (2023) 20:57. doi: 10.1186/s12984-023-01186-9, PMID: 37131238 PMC10155325

[8] Fujii C Wakizaka N Araki Y Tashiro K Endou M. Video analysis of safety and reproducibility issues with the timed up-and-go test applied to patients with Parkinson’s disease. Disabil Rehabil Assist Technol. (2021) 17:801–6. doi: 10.1080/17483107.2020.1817990, PMID: 34171200

[9] Postuma RB Berg D Stern M Poewe W Olanow CW Oertel W. MDS clinical diagnostic criteria for Parkinson's disease. Mov Disord. (2015) 30:1591–601. doi: 10.1002/mds.26424, PMID: 26474316

[10] Tomlinson CL Stowe R Patel S Rick C Gray R Clarke CE. Systematic review of levodopa dose equivalency reporting in Parkinson's disease. Mov Disord. (2010) 25:2649–53. doi: 10.1002/mds.23429, PMID: 21069833

[11] Saranza G Lang AE. Levodopa challenge test: indications, protocol, and guide. J Neurol. (2020) 268:3135–43. doi: 10.1007/s00415-020-09810-7, PMID: 32333167

[12] Wu T Hallett M Chan P. Motor automaticity in Parkinson's disease. Neurobiol Dis. (2015) 82:226–34. doi: 10.1016/j.nbd.2015.06.014, PMID: 26102020 PMC5565272

[13] Takakusaki K. Functional neuroanatomy for posture and gait control. J Movement Disorders. (2017) 10:1–17. doi: 10.14802/jmd.16062, PMID: 28122432 PMC5288669

[14] Peterson DS Horak FB. Neural control of walking in people with parkinsonism. Physiology. (2016) 31:95–107. doi: 10.1152/physiol.00034.2015, PMID: 26889015 PMC4888974

[15] Popova M Messé A Gulberti A Gerloff C Pötter-Nerger M Hilgetag CC. The effect of deep brain stimulation on cortico-subcortical networks in Parkinson’s disease patients with freezing of gait: exhaustive exploration of a basic model. Network Neurosci. (2024) 8:926–45. doi: 10.1162/netn_a_00376, PMID: 39355431 PMC11424038

[16] Avanzino L Lagravinese G Abbruzzese G Pelosin E. Relationships between gait and emotion in Parkinson’s disease: a narrative review. Gait Posture. (2018) 65:57–64. doi: 10.1016/j.gaitpost.2018.06.171, PMID: 30558947

[17] de Almeida FO Ugrinowitsch C Brito LC Milliato A Marquesini R Moreira-Neto A. Poor sleep quality is associated with cognitive, mobility, and anxiety disability that underlie freezing of gait in Parkinson’s disease. Gait Posture. (2021) 85:157–63. doi: 10.1016/j.gaitpost.2021.01.026, PMID: 33578308

[18] Morris R Martini DN Madhyastha T Kelly VE Grabowski TJ Nutt J. Overview of the cholinergic contribution to gait, balance and falls in Parkinson's disease. Parkinsonism Relat Disord. (2019) 63:20–30. doi: 10.1016/j.parkreldis.2019.02.017, PMID: 30796007 PMC6682416

[19] Gilat M. Lígia Silva de Lima a, Bloem BR, Shine JM, Nonnekes J, Lewis SJG: freezing of gait: promising avenues for future treatment. Parkinsonism Relat Disord. (2018) 52:7–16. doi: 10.1016/j.parkreldis.2018.03.009, PMID: 29550375

[20] Kwok JYY Smith R Chan LML Lam LCC Fong DYT Choi EPH. Managing freezing of gait in Parkinson’s disease: a systematic review and network meta-analysis. J Neurol. (2022) 269:3310–24. doi: 10.1007/s00415-022-11031-z 35244766

